# *Callistemon viminalis* leaf extract phytochemicals modified silver–ruthenium bimetallic zinc oxide nanocomposite biosynthesis: application on nanocoating photocatalytic *Escherichia coli* disinfection

**DOI:** 10.1039/d4ra01355g

**Published:** 2024-04-05

**Authors:** Pankaj Kumar Jha, Tunyakamon Jaidumrong, Dinesh Rokaya, Chitchamai Ovatlarnporn

**Affiliations:** a Department of Pharmaceutical Chemistry, Faculty of Pharmaceutical Sciences, Prince of Songkla University Hat Yai Songkhla 90110 Thailand nanoscience.jha.pankaj@gmail.com; b Faculty of Environmental Management, Prince of Songkla University Hat Yai Songkhla 90110 Thailand tunya.envimicro@gmail.com; c Department of Prosthodontics, Faculty of Dentistry, Zarqa University Zarqa 13110 Jordan dineshrokaya115@hotmail.com; d Drug Delivery System Excellence Center, Prince of Songkla University Hat Yai Songkhla 90110 Thailand nanoscience.jha.pankaj@gmail.com

## Abstract

Antibiotics are of great interest due to antibiotic-resistant problems around the globe due to bacterial resistance to conventional antibiotics. In this study, a novel green biosynthesis of silver–ruthenium bimetallic zinc oxide nanocomposite using *Callistemon viminalis* leaf extract as a reducing agent using zinc nitrate hexahydrate, silver nitrate, and ruthenium(iii) chloride as capping agents was reported. The results demonstrated that the surface morphology of the prepared bimetallic nanocomposite by scanning electron microscopy was hexagonal in shape for zinc nanoparticle, rectangular in shape for silver nanoparticle, and tetragonal in shape for ruthenium nanoparticle, having an average surface size 25, 35, and 55 nm, respectively. Fourier transform infrared analysis confirmed the presence of compounds containing alkene, halo-, sulfoxide, phenol, nitro-, phenyl-ester, carboxylic acid, amines, and alcohols which act as functional groups attached to the surface of nanocomposites. Results from X-ray diffraction analysis found 81.12% crystallinity and hexagonal structure of zinc nanoparticles, rectangular structure of silver nanoparticles, and tetragonal structure of ruthenium nanoparticles, which are also similar to the results from transmission electron microscopy analysis. The average size distribution by dynamic light scattering of silver–ruthenium bimetallic zinc oxide nanocomposite was 255 nm, which confirms the biosynthesis of non-uniform size. Photo-disinfection activity of a silver–ruthenium bimetallic zinc oxide nanocomposite against *Escherichia coli* bacteria isolated from hospital wastewater under dark and ultraviolet-A irradiation conditions was observed. The antibacterial activity was calculated at 2.42704239, ensuring the silver–ruthenium bimetallic zinc oxide nanomaterials have photo-disinfection properties. The results from this study revealed that the developed novel antibacterial nanocomposite of silver–ruthenium bimetallic zinc oxide is useful in nanocoating photocatalytic *Escherichia coli* disinfection and can be applied to disinfect surfaces.

## Abstract 2

*Callistemon viminalis* leaf extract, Ag–Ru/ZnO bimetallic nanocomposite, nanocoating UVA light photo-disinfection, antibacterial application.

## Graphical abstract

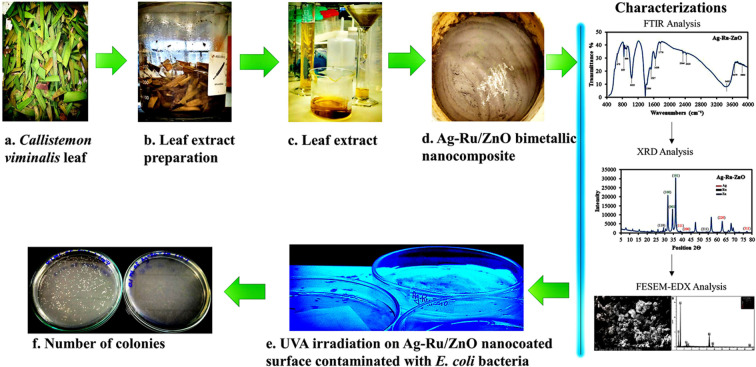

## 1. Introduction

The antibiotic-resistant problem is the biggest threat in the healthcare sector.^1^ The problem arises due to overuse, misuse of antibiotics, and microbial gene mutation. New species of antibiotic-resistant bacteria could be due to microbial evolution by external influences like-the environment and chemicals, leading to a microbial threat to human health.^2^ There are various sources of contamination, such as drainage waste, household wastewater, and industrial waste, that can cause microbial resistance. However, the main source of microbial contamination in hospital surroundings is wastewater, which is highly microbially contaminated due to more infected patients' activities.^3^ Hospital wastewater is normally treated by wastewater treatment plants to disinfect contaminated water using commercially available antibiotics, but the antibiotic-resistant problem becomes a major cause of difficulty in the wastewater disinfection process.^4^*Escherichia coli* is one of the most common microbial contaminants found in hospital wastewater. Contact with *E. coli* may lead to infectious diseases such as skin infection, diarrhea, *etc.*^5^

In developing countries, the health hygiene of people is very poor due to poverty and a lack of health facilities such as hospital hygiene management, wastewater treatment and management, and vaccination. This may result in several healthcare threats, like infections, wounds, and various diseases. In developed countries, although there is proper healthcare management, microbial resistance and its associated problems are major threats to healthcare management.^6^ In search of new formulations and techniques to overcome microbial resistance problems, newly developed formulations and materials are used in healthcare applications such as nanocoating for surface disinfection and wastewater management. Nanotechnology is an emerging field with its various sub-branches like nano-biomedicine, nano-chemistry, nanobiology, nano-systems, *etc.*, where various biological applications are done by synthesizing nanoscale particles.^7^ In this nanoscale particles synthesis process, there are different techniques, such as physical and chemical methods. Physical methods are physical vapor deposition (PVD), ball milling, pulse laser ablation (PLA), and chemical methods are chemical vapor deposition (CVD), chemicals co-precipitation, which are hazardous to the environment and costly on an economic level. However, green synthesis or biosynthesis method for nanoparticles is a keen interest in material processing and formulations due to its ecofriendly and being commonly available in our surroundings.^8,9^

Green synthesis is also very important on the surface of the nanomaterial functionalization process.^10,11^ Various types of functional groups from natural products, such as phenolic, ketones, carboxylic, alkanes, alkenes, *etc.*, can be utilized in attachment to the surface of nanomaterials.^12,13^ Many compounds from natural products have high stability and biological functions such as antimicrobial, anti-inflammatory, *etc.*, which may assist the biological properties of the nanomaterial when they are incorporated into the preparation process. The chemical functional groups of bio-material play a vital role in reducing bulk materials into nanoscale materials and encapsulating them to make them stable, which is very interesting in nanomaterials during biosynthesis process.^14^ In contrast to the chemical and physical methods, which are not reliable for synthesizing nanomaterials due to chemical hazards and risks in operation.^15^ In biological or green synthesis methods, plant parts such as leaves, roots, stems, and biological wastes are commonly used to synthesize the nanomaterials.^16^ In this research, leaves from the *Callistemon viminalis* plant were taken to perform biosynthesis process. *C. viminalis* is a tropical plant commonly found in Asian countries and has been reported to have various medicinal applications such as antibacterial, antifungal, antioxidant, and other pharmaceutical and insecticidal properties.^17^ Its leaves were reported to contain 1,8-cineole, α-pinene, α-terpineol, terpinen-4-ol, 3-*O*-α-l-arabinopyranoside hederagenin, hederagenin 3-*O*-β-glucopyranosyl-(1→2)-β-d-xylopyranoside, betulinic acid, *etc.*,^18^ which acts as a reducing agent and capping agent during nanoparticles biosynthesis process,^19^ the extract has antibacterial properties.^20^ Thus, this research aims to synthesize silver–ruthenium bimetallic zinc oxide nanocomposite using leaf extract of *Callistemon viminalis* and determine its application on nanocoating photocatalytic *E. coli* disinfection.

## 2. Results

### 2.1 Biosynthesis of silver–ruthenium bimetallic zinc oxide nanocomposites (Ag–Ru/ZnO bimetallic nanocomposite)

**Fig. 1:**
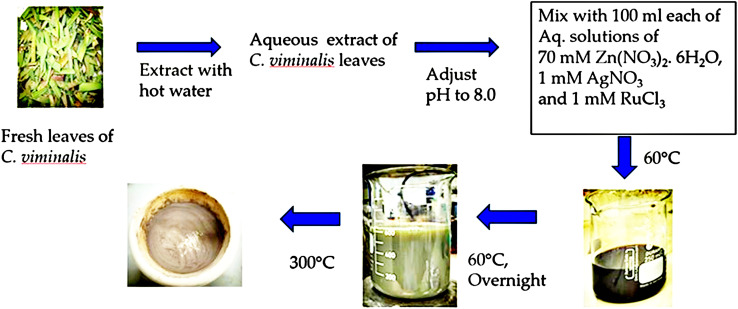
Biosynthesis of silver–ruthenium bimetallic zinc oxide nanocomposites.

The synthesis process of Ag–Ru/ZnO bimetallic nanocomposite (Fig. 1) was performed similarly to that previously reported by Jha *et al.* (2021).^21^ Fresh leaves of *C. viminalis* were collected, washed, and extracted from 30 g by mixing with 250 mL hot water and filtered through a Whatman No. 1 filter paper resulting in a light-yellow solution. The 10 mL solution of leaf extract was further utilized in the nanocomposite synthesis in aqueous alkali (pH 8) condition. Inorganic precursors used in the synthesis process comprise solutions of zinc nitrate hexahydrate, silver nitrate, and ruthenium(iii) chloride in water. The synthesis process was performed at 60 °C in the dark after mixing the solution of leaf extract with all metal precursor solutions. The vigorous stirring continued until the solution's colour changed from dark black to light brown, confirming the nanocomposites were successfully achieved. The final dried powder of Ag–Ru/ZnO bimetallic nanocomposite was finally obtained by annealing at 300 °C for 3 hours to obtain a pure ultrafine form of nanomaterials attached with functional groups as a dark grey powder, where nanomaterial crystallinity, phase transition, and photocatalytic properties increase.

### 2.2 Ultraviolet visible and band gap energy analysis

**Fig. 2:**
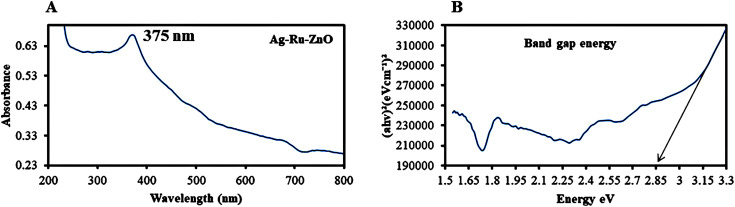
(A) Ultraviolet visible absorbance peak of Ag–Ru/ZnO bimetallic nanocomposite at 375 nm and (B) band gap energy of 2.85 eV.

The ultraviolet visible (UV-vis) absorbance (Fig. 2) of Ag–Ru/ZnO nanocomposite was observed at 375 nm, confirming decreasing frequency, which is redshift as shown in Fig. 2A, and from the eqn (2), band gap energy was at calculated 2.85 eV as shown in Fig. 2B, which is similar and better than the report of Gurgur *et al.* (2020).^22^

### 2.3 Field emission scanning electron microscopy-energy dispersive X-ray analysis

**Fig. 3:**
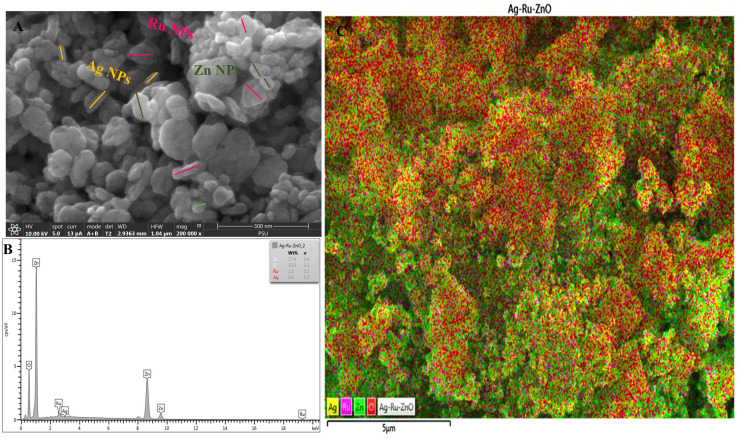
(A) Field emission scanning electron microscopic analysis showing non-uniform size of Ag–Ru/ZnO bimetallic nanocomposite, hexagonal shape of ZnO nanoparticles, rectangular shape of Ag nanoparticles, and tetragonal shape of Ru nanoparticles (B) energy dispersive X-ray (EDX) analysis showing elemental weight percentage present in the Ag–Ru/ZnO bimetallic nanocomposite. (C) Picture from the EDX mapping.

Field emission scanning electron microscopy (FESEM, Fig. 3) analysis showed the average surface size of small zinc (Zn) nanoparticles of 25 nm to be hexagonal in shape. The surface size of small silver (Ag) nanoparticles had an average size of 35 nm with rectangular in shape. Similarly, surface size of small size ruthenium (Ru) nanoparticle displayed tetragonal shape with an average size of 55 nm as shown in Fig. 3A. However, non-uniform surface and size characteristics of zinc oxide, silver, and ruthenium were observed, confirming that each nanoparticle has different sizes, such as large, medium, and small. Energy dispersive X-ray (EDX) analysis has shown a weight percentage of 77.4% of Zn nanoparticles, 0.4% of Ag nanoparticles, and 1.3% of Ru nanoparticles, and 20.9% of oxygen (O), with all elements well distributed as shown in Fig. 3B and C, in contrast, nanomaterials arrangements are different in comparison to the report of Pragati *et al.* (2014).^23^

### 2.4 Dynamic light scattering analysis

**Fig. 4:**
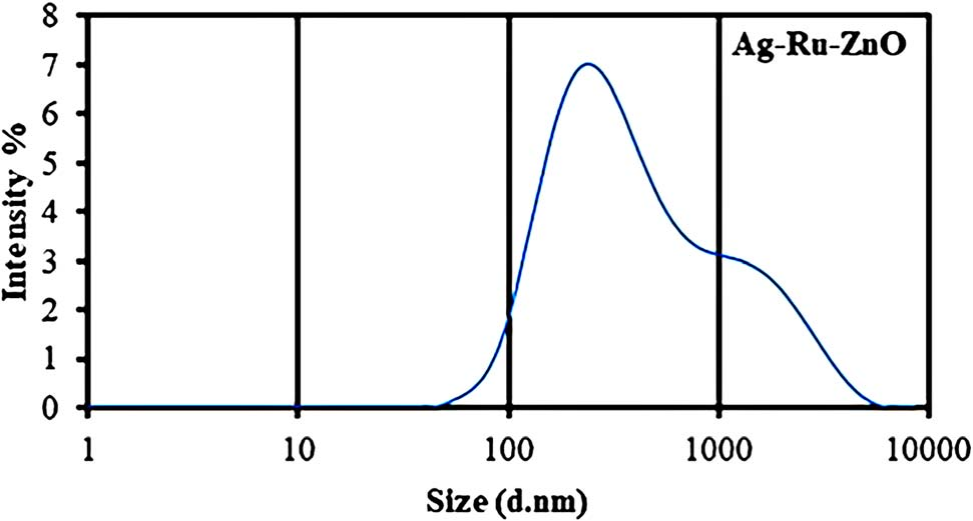
Dynamic light scattering analysis of Ag–Ru/ZnO bimetallic nanocomposite showing an average size distribution of 255 nm (PDI = 0.54).

Dynamic light scattering (DLS) is photon correlation spectroscopy and one of the most popular methods used to determine the size of nanomaterials.^24^ DLS analysis of the obtained Ag–Ru/ZnO bimetallic nanocomposite (Fig. 4) showed an average size distribution of 255 nm in the obtained Ag–Ru/ZnO bimetallic nanocomposite with a polydispersity index of 0.54, confirming non-uniform size. The non-uniform size may be due to some aggregation of the composite during the annealing process. Moreover, the sizes of the nanomaterials by determined DLS analysis are results of the random motion due to their kinetic energy. The variation in sizes could also be due to the different shapes of the nanomaterials.

### 2.5 Transmission electron microscopy analysis

**Fig. 5:**
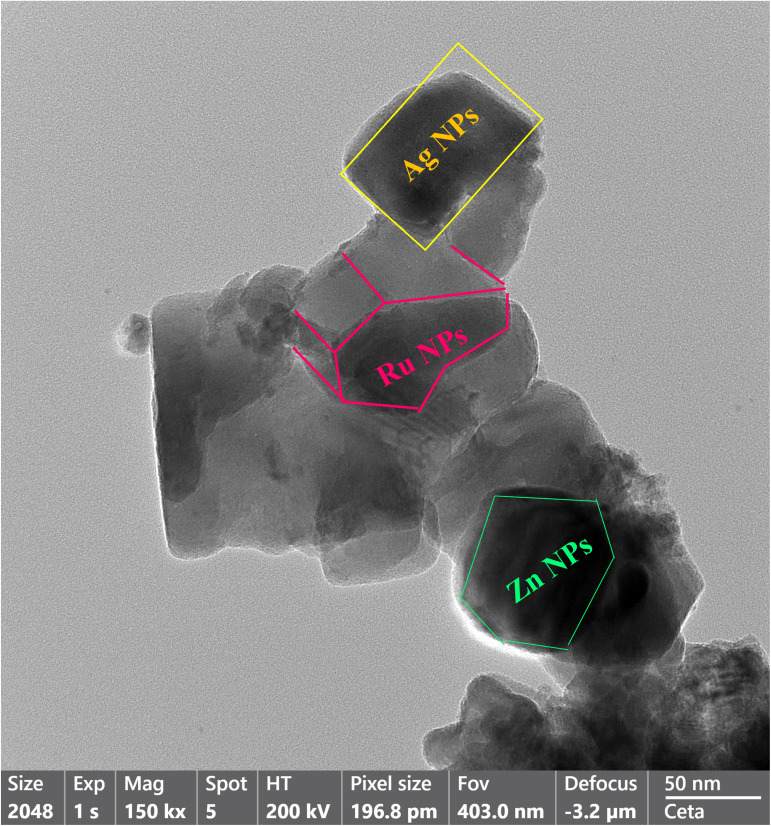
Transmission electron microscopy analysis structure confirmation of silver having a rectangular structure, ruthenium having tetragonal structure, and zinc oxide having hexagonal structure.

The study on the structure of the Ag–Ru/ZnO bimetallic nanocomposite was performed using transmission electron microscopy (TEM). The obtained transmission electron micrographs (Fig. 5) demonstrated the hexagonal structure of Zn nanoparticles, rectangle structure of Ag nanoparticles, and the tetragonal structure of Ru nanoparticles. The average diameter of a nanoparticle is ∼20–50 nm in crystalline form, in the same manner as reported by Bhunia *et al.* (2015).^25^

### 2.6 X-ray diffraction analysis

**Fig. 6:**
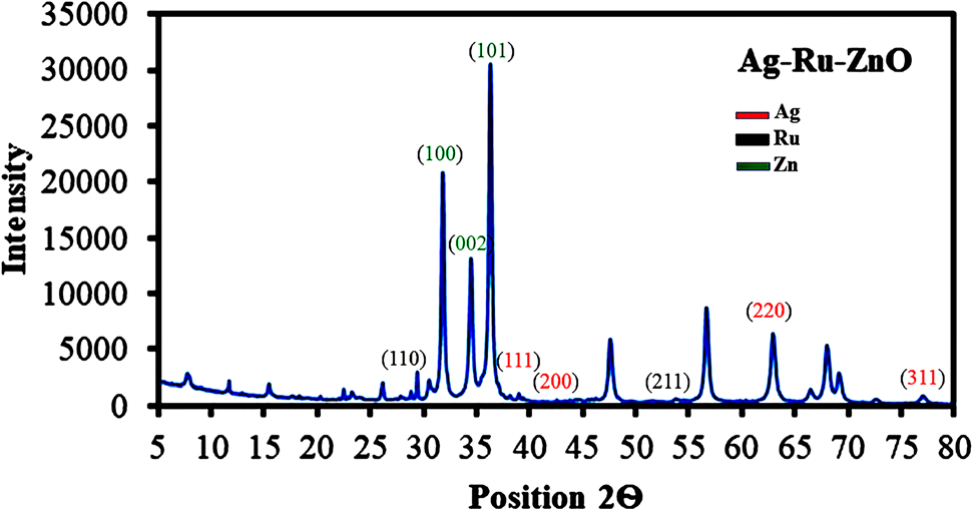
X-ray diffraction analysis confirmed the synthesis of Ag–Ru/ZnO bimetallic nanocomposite.

The X-ray diffraction (XRD) pattern (Fig. 6) of the Ag–Ru/ZnO bimetallic nanocomposite shows peaks and muller indices (*h*, *k*, *l*) value at positions (2*θ*) 31.65° (1 0 0), 34.32° (0 0 2), and 36° (1 0 1), indicating hexagonal crystal structure of zinc nanoparticles. XRD peaks and *h*, *k*, *l* values of Ag nanoparticles were observed at 38° (1 1 1), 44.6° (2 0 0), and 64.1° (2 2 0), indicating a rectangular crystal structure. Similarly, Ru nanoparticles were observed at 28° (1 1 0) and 54.2° (2 1 1), indicating a tetragonal crystal structure. The metallic crystal size calculated by eqn (2) was found to be 28 nm for ZnO nanoparticles, 59 nm for Ag nanoparticles, and 72 nm for Ru nanoparticles. The percentage of crystallinity was found to be 81.12%, similar to the published data of Bushell *et al.* (2020).^26^

### 2.7 Fourier transform infrared analysis

**Fig. 7:**
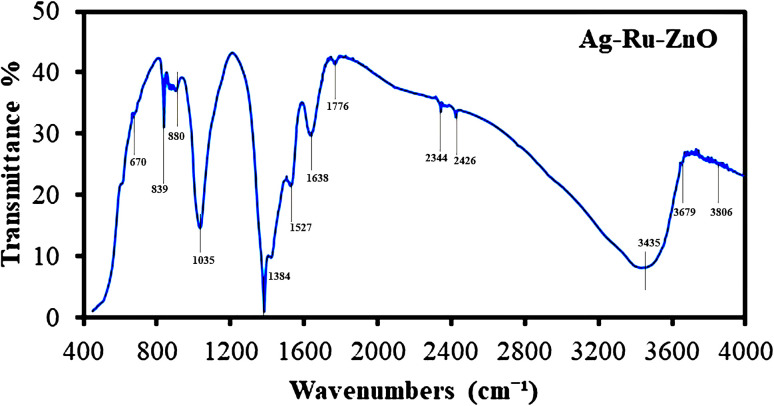
Fourier transform infrared spectrum of Ag–Ru/ZnO bimetallic nanocomposite (KBr).

Fourier transform infrared can be used to identify functional groups that attach on the surface of the obtained nanomaterials.^27^ The FTIR transmission spectrum of Ag–Ru/ZnO bimetallic nanocomposite (Fig. 7) displays peaks at wavenumbers (cm^−1^) 670, 839, 880, 1035, 1384, 1527, 1638, 1776, 2344, 2426, 3435, 3679, and 3806 indicating the presence of alkene, halo compound, sulfoxide, phenol, nitro compound, phenyl-ester, carboxylic acid, amines, primary alcohols, and alcohols, respectively. Moreover, C=C bending, C–Cl stretching, S=O stretching, O–H bending, and N–O stretching were noticed in the spectrum, confirming the functional groups contained in the extract of *C. viminalis* that may be attached on the surface of the nanomaterials. In previous studies, *C. viminalis* showed the presence of phenols, alcohol, carboxylic acid, ketones, and amines which was earlier reported in Jha *et al.* (2023).^28^

### 2.8 Photocatalytic nanocoating disinfection in antibacterial application

A liter of wastewater was filtered through a nitrocellulose filter (pore size 0.22 μm). The filtrate was taken into 500 mL of nutrient broth media and incubated for 24 hours. After incubation, the nutrient broth media with culture bacteria was streaked on MacConkey agar and incubated for 24 hours. The pink colonies were collected from the MacConkey agar and re-streaked on eosin methylene blue (EMB) agar. *E. coli* bacteria in the metallic green sheen colonies were observed. A single colony of *E. coli* was picked, and it was subcultured in nutrient agar media. In the meantime, 25 g of Luria Bertani (LB) broth media was suspended in 1000 mL of deionized water and autoclaved at 121 °C for 15 minutes to sterilize. After autoclaving, LB broth was cooled and poured into the different round-bottom flasks inside the laminar airflow, and *E. coli* bacteria were suspended in it similar to Behesti Maal *et al.* (2015).^29^

Two percent (2%), *i.e.*, 20 mg mL^−1^ of nanomaterials suspension was prepared. Whatmann No. 1 filter papers were cut into 5 cm × 5 cm sizes and 1 mL of the obtained silver–ruthenium bimetallic zinc oxide (Ag–Ru/ZnO) nanocomposite was poured on the paper and further annealed at 100 °C for 30 minutes to make it sterile. One milliliter of hospital wastewater containing *E. coli* (4.04 × 10^4^ CFU mL^−1^) was poured on the nanocoated paper and covered by a glass coverslip to perform nanocoating experiment. One paper was free of nanomaterials as a control sample. Here, for the bacteria respiration oxygen inlet and outlet pipe were connected to the light-box to maintain the internal environment of light box chamber. In the first step, both control and nanocoated samples were incubated under dark conditions for 8 hours and then irradiated with 18 W/UV-A for another 8 hours under light condition, maintaining the distance between samples and lamp at 19 cm. Bacterial samples were recovered from the edge of the cover slip by using a micro-pipette. Spiral plating was performed to count *E. coli* bacterial colonies before and after irradiation to understand bacterial depletion under different conditions by culturing 100 μL of bacterial samples on nutrient agar media using L shape inoculating loop, similar to the method of Gkana *et al.* (2017).^30^

**Fig. 8:**
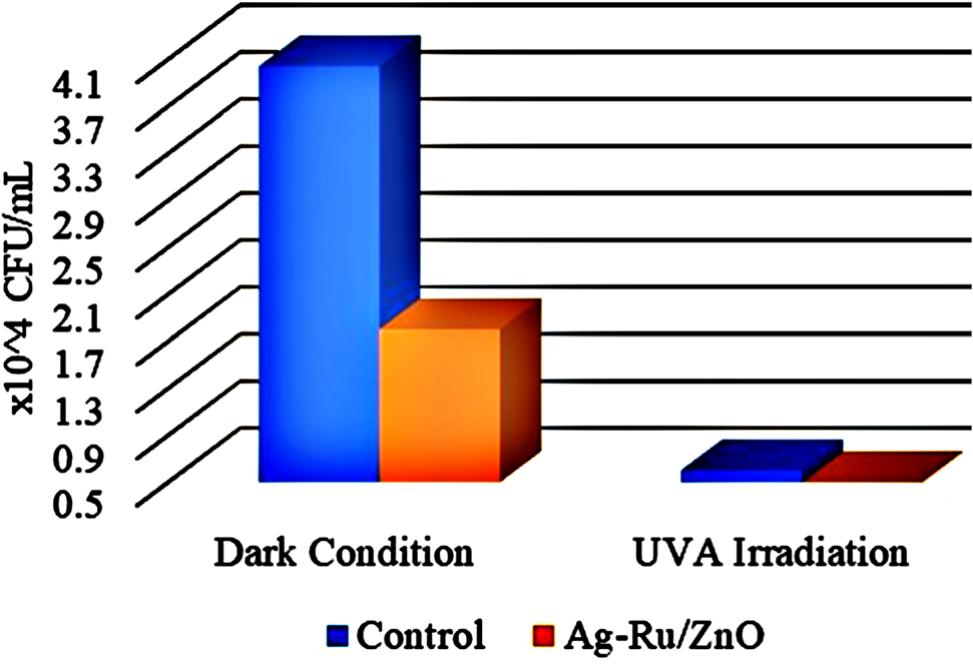
CFU mL^−1^ of control, and Ag–Ru/ZnO nanocomposite under 8 hours of dark conditions, and 8 hours of UV-A light irradiation.

As shown in Fig. 8, colony formation unit (CFU mL^−1^) under dark and light conditions in control have higher in numbers in comparison with Ag–Ru/ZnO bimetallic nanocomposite coatings. The antibacterial activity was determined using eqn (1) similar to Xie *et al.* (2011)^31^ and the results are summarized in Table 1.1
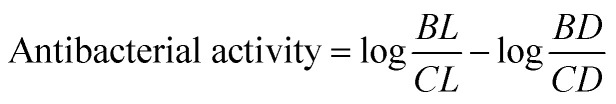
where *L* is light condition and *D* is dark condition, *B* is several bacteria in the non-photocatalyst sample, and *C* is several bacteria in the nanocoated Ag–Ru/ZnO bimetallic nanocomposite.

**Table 1:** Number of recovered bacteria in CFU mL^−1^ before and after irradiation

Samples	Initial bacteria before testing 4.04 × 10^4^ (CFU mL^−1^)	
	Recovered bacteria after 8 hours in dark condition (CFU mL^−1^)	Recovered bacteria after 8 hours of irradiation (CFU mL^−1^)
Control	4.04 × 10^4^	0.6 × 10^4^
Ag–Ru/ZnO	1.80 × 10^4^	0.001 × 10^4^

Table 1 demonstrated the antibacterial activities of the Ag–Ru/ZnO bimetallic nanocomposite against wastewater contaminated bacteria. The initial bacterial counts before testing were 4.04 × 10^4^ CFU mL^−1^. The control experiments found that when there was no treatment with Ag–Ru/ZnO bimetallic nanocomposite in the dark condition, the amount of bacterial count remained at the same value showing a stationary phase. However, in another control group (no nanocomposite), but in the presence of UV-A light, the amount of bacterial count was reduced to 0.6 × 10^4^ CFU mL^−1^, indicating that UV-A itself has antibacterial activity. In contrast to the test groups by treated with the Ag–Ru/ZnO bimetallic nanocomposite both without UV-A irradiation and with UV-A irradiation, the number of bacterial counts was significantly reduced when compared to the initial count. Interestingly, when the treatment was performed by the addition of the nanocomposites together with UV-A irradiation, a significant reduction in comparison to the same treatment but no UV-A irradiation was observed. The amount of bacterial count was reduced from the initial amount about 1800 times in the treatment group in the dark. Bacterial counts in the treatment group with light irradiation were in 4040 times in the non-treatment group compared to the treatment group with light irradiation. The antibacterial activity of the developed nanocomposite was calculated using eqn (1) and resulting in a value of 2.42704239. The significance observed at 95% confident of dark condition was calculated to be significance value (*P*) = 0.0000, which is statistically significant with 55.45% of Ag–Ru/ZnO nanocomposite antibacterial rate comparison with 0% of control antibacterial rate. Similarly, the significance observed at 95% confident of light condition was calculated to be significance value (*P*) = 0.0000, which is statistically significant with 99.94% of Ag–Ru/ZnO nanocomposite antibacterial rate comparison with 85.15% of control antibacterial rate.

## 3. Discussion

Silver–ruthenium bimetallic zinc oxide nanocomposite was obtained by biosynthesis using a green method. None of the organic solvents were utilized in the synthesis process. The obtained Ag–Ru/ZnO bimetallic nanocomposite displayed maximum UV-vis absorbance at 375 nm, indicating the success of the nanomaterial's products from this process. The narrow energy band gap of the obtained nanocomposite was 2.85 eV which is beneficial for electrostatic interaction between divalent Zn^2+^ ions in Ag–Ru/ZnO nanocomposite. This interaction would effectively increase the vacancy defects in Ag–Ru/ZnO nanocomposite crystals, therefore enhancing the electron transition from the valence band to the conduction band as well as decreasing the charge carrier separation, which is helpful for photoactivity disinfection properties.^32^ Scanning electron microscopy analysis revealed the surface morphology of ZnO nanoparticles to be hexagonal shape, silver nanoparticles to be in rectangular shape, and ruthenium nanoparticles to be in tetragonal shape. Similarly, dynamic light scattering analysis gave an average size distribution of 255 nm with a high polydispersity index, which may be due to the aggregation of the nanomaterial during the annealing process. X-ray diffraction has confirmed the crystallinity of the obtained nanocomposites, which having an 81.12% crystallinity containing metals from the highest to the lowest: Zn, Ag, and Ru, respectively. Transmission electron microscopy analysis has confirmed the structure of nanomaterials, similar to SEM and XRD analysis. The information from the FT-IR spectrum confirmed the presence of several functional groups such as alkene, halo compound, alkane, sulfoxide, phenol, nitro compound, phenyl-ester, carboxylic acid, amines, and alcohols attached on the surface of silver–ruthenium bimetallic zinc oxide nanocomposite. Those functional groups were derived from the extract of *C. viminalis* leaf. The antibacterial activity value of Ag–Ru/ZnO bimetallic nanocomposite with light illumination was 2.42704239 times higher than in the dark condition, which may be due to the UV-A light absorption properties of the attached functional groups on the surface of Ag–Ru/ZnO nanocomposite. In contrast, UV-A light irradiation on Ag–Ru/ZnO nanocomposite activates electron transfers from the valence band to the conduction band, inhibiting redox oxygen species (ROS) binding with *E. coli* and leading to bacterial depletion. UV-A light strikes the surface of the nanocomposite to generate excited band gap energy, which promotes the formation of reactive oxygen species (ROS) as an antibacterial agent and shows antibacterial properties at the nano level which is already explained the nanomaterials' ROS mechanism by Ghobani *et al.* (2018), Li *et al.* (2012), Joe *et al.* (2018),^33–35^ meanwhile, for Ag–Ru/ZnO nanocomposites' an experimental study on ROS mechanism is required in future. The control sample (Ag–Ru/ZnO bimetallic nanocomposite without UV-A) did not have any disinfection properties against *E. coli* compared to that of the bimetallic nanocomposite sample under UV-A irradiation. Even though the control sample was found to have an inactivation tendency under UV-A irradiation, but in comparison with the Ag–Ru/ZnO bimetallic nanocomposite, it has no capability to reach the activation level in the ROS (superoxide radical, hydroxyl radical, and singlet oxygen) production process. Ag–Ru/ZnO bimetallic nanocomposite has a higher inactivation tendency under UV-A irradiation, leading to a higher amount of ROS production. Irradiated Ag–Ru/ZnO bimetallic nanocomposite can induce common oxidative damage in bacteria, leading to the production of reactive oxROS and DNA, protein, and lipid damage which results in cell death.^36,37^ In this study, photo-disinfection showed potential applications of bimetallic nanocomposite for disinfection purposes such as surface cleaning and photocatalytic disinfection at wastewater treatment plants to cope with antibiotic-resistant problems but further cytotoxicity studies are required. Also, it could be applicable in household antimicrobial cleaning and coating on the surface for instrumental disinfection.

## 4. Materials and methods

### 4.1 Materials

*Callistemon viminalis* leaf was brought from Agricultural Park, Hat Yai, Songkhla-90110, Thailand. Zinc nitrate hexahydrate and Ruthenium(iii) chloride hydrate were purchased from Sigma Aldrich, Singapore. Silver nitrate and sodium hydroxide were purchased from Loba Chem Pvt. Ltd., Thailand. Nutrient broth media, Nutrient Agar Media, Muller Hilton Agar Media, Eosin Methylene Blue (EMB) Media, and Luria Bertani (LB) Media were brought from HiMedia Laboratories Pvt. Ltd., Mumbai, India. MacConkey Agar was purchased from Becton, Dickinson, and Company, Sparks, MD 21152, USA.

### 4.2 Methods

#### 4.2.1 Preparation of *C. viminalis* leaf extract

Thirty grams of leaves were washed with de-ionized water and cut into small pieces. After cutting into small pieces, it was boiled with 250 mL of de-ionized water for 20 minutes at 60 °C, cooled, and filtered with Whatman No. 1 filter paper, similar to Jain and Jain (2017).^38^

#### 4.2.2 Biosynthesis of silver–ruthenium bimetallic zinc oxide nanocomposites

A hundred milliliter solution of 70 mM zinc nitrate hexahydrate, 1 mM silver nitrate solution (100 mL), and 1 mM ruthenium(iii) chlorite solution (100 mL) were prepared. A mixture of zinc precursor solution, silver nitrate solution, and ruthenium solution was prepared with 10 mL of the leaf extract solution. Drops of 1 M sodium hydroxide (NaOH) were added to make pH 8, and it was also heated at 60 °C under vigorous stirring until color changed from dark black to light brown, followed by annealing at 300 °C for 3 hours to collect powder similar to Jha *et al.* (2023).^39^

#### 4.2.3 Photocatalytic nanocoating disinfection studies

**Fig. 9:**
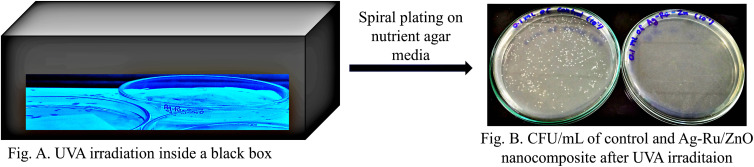
Photo-disinfection studies method by UVA light irradiation.

Wastewater was collected from the wastewater treatment plant of Songklanagarind Hospital, Kho Hong, Hat Yai, Songkhla, Thailand. The adhesion method is the process of attaching surfactant to the surface. The glass adhesion method for nanocoating photo-disinfection activity was performed using 2% of nanomaterials concentration (*i.e.*, 20 mg mL^−1^) and hospital wastewater *E. coli* bacteria concentration of 4.04 × 10^4^ CFU mL^−1^ (Fig. 9) similar to Chatterjee *et al.* (2023).^40^

### 4.3 Characterizations

Ultraviolet visible analysis was conducted at wavelengths from 200 to 800 nm using UV-visible spectroscopy model number DR 6000 (Environmental Science Company Limited). Band gap energy was calculated using the following TAUC formula (eqn (2)), similar to Makula *et al.* (2018):^41^

2 *αhγ* = *A*[*hγ* − *E*_gap_]^*n*^

where *α* is the linear absorption coefficient, *h* is Planck's constant, *γ* is the frequency of light, *A* is a proportionality constant, *E*_gap_ is the band gap energy, and *n* is a numerical value depending upon the transition. A graph was plotted between *αhγ vs. hγ* and the band gap energy was estimated.

The surface morphology and elemental compositions were examined using FE-SEM Apreo-EDX. TEM analysis was performed using an electron probe X-ray microanalyzer JXA 8900R. The functional group attached to the nanoparticle surface was analyzed by the KBr pallet technique using a Fourier Transform Infrared Spectrometer (VERTEX 70, Bruker, Bremen, Germany). XRD Empyrean was used to identify crystallinity and phase identification. The average crystal size was calculated using Scherer's equation, similar to Ali *et al.* (2013):^42^3
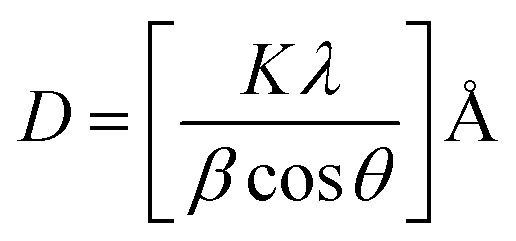
where *D* is the average crystallite diameter in Angstrom, *K* is the Scherrer constant, *λ* is the wavelength of X-ray, *i.e.*, 1.5406 Å CuKα radiation, *θ* is the Braggs' angle, and *β* is the full width at half maximum intensity of diffraction peak. Nanomaterials particle size distribution were analyzed using dynamic light scattering (DLS), Malvern Panalytical, Zetasizer version 7.13.

## Sample availability

Samples of the compounds are available from the authors.

## Data availability

Furnish upon request.

## Author contributions

Conceptualization, experimental, methodology design, and writing – original draft preparation, supervision, P.·K. J.; technical support, T. J.; data checking, proofreading, D. R.; partial supervision, final proofreading and correction, C. O. All authors have read and agreed to the published version of the manuscript.

## Conflicts of interest

The authors declare no conflict of interest.

## Supplementary Material
